# Does time spent upright moderate the influence of a weighted vest on change in bone mineral density during weight loss among older adults? A secondary analysis of the INVEST in bone health randomized controlled trial

**DOI:** 10.3389/fragi.2026.1729001

**Published:** 2026-02-11

**Authors:** Jason Fanning, Daniel P. Beavers, Shirley M. Bluethmann, S. Delanie Lynch, Ashley A. Weaver, Cassidy Guida, Kristen M. Beavers

**Affiliations:** 1 Department of Health and Exercise Science, Wake Forest University, Winston-Salem, NC, United States; 2 Department of Statistical Sciences, Wake Forest University, Winston-Salem, NC, United States; 3 Department of Social Sciences and Health Policy, Wake Forest School of Medicine, Winston-Salem, NC, United States; 4 Department of Biomedical Engineering, Wake Forest School of Medicine, Winston-Salem, NC, United States; 5 Department of Internal Medicine, Wake Forest School of Medicine, Winston-Salem, NC, United States

**Keywords:** accelerometry, aging, bone, exercise, sedentary behavior, weight loss

## Abstract

**Introduction:**

The INVEST in Bone Health randomized controlled trial examined whether 1 year of weight loss paired with resistance training (WL+RT) or weighted vest use (WL+VEST) sustained bone mineral density (BMD) at the hip better than weight loss alone (WL). All groups lost similar amounts of body weight, but neither those wearing the vest nor those engaging in structured resistance training retained greater bone density relative to those in the weight loss-only condition. One possible reason for the absence of a group difference may be that the bone-sparing benefits of the weighted vest may relate to the amount of time one spends standing and therefore exposed to additional loading Purpose: The purpose of this secondary analysis was to determine whether the time an individual spent upright moderated the effect of the intervention on BMD at the hip.

**Methods:**

Older adults (mean age 66.9 ± 4.8 years) were eligible if they were living with obesity or who were both overweight with an indication for weight loss. Participants were randomized to one of the three WL interventions. Participants completed quantitative computed tomography (CT) and dual energy x-ray absorptiometry (DXA) assessments of hip BMD and wore an ActivPAL accelerometer to measure upright time for 1 week at baseline, 6, and 12 months.

**Results:**

In total 131 participants had sufficient DXA data and 132 and sufficient CT data for inclusion in linear mixed effects models. The model for DXA-derived hip areal BMD (aBMD) revealed a significant interaction between upright time and group assignment (p = 0.023) such that upright time was positively associated with baseline-adjusted change in aBMD in WL+VEST, but the opposite was true for WL (p = 0.009). The relationship between upright time and change in aBMD likewise differed significantly between WL+RT and WL (p = 0.043), as WL+RT demonstrated a less negative relationship than did WL. There were no significant interactions between group assignment and upright time for CT-derived measures of BMD.

**Conclusion:**

These results suggest a need for research investigating the efficacy of a weighted vest intervention paired with a focus on improving daily upright time for sustaining bone health among older adults as they lose weight.

## 1 Introduction

Obesity in older adults is associated with poorer quality of life and physical function and greater risk of frailty, and these outcomes tend to improve with intentional weight loss (Gill et al., 2015; Villareal et al., 2005). Still, many clinicians and health promotion professionals struggle to recommend weight reduction in this population, as approximately one-quarter of lost weight is fat-free mass (Heymsfield et al., 2014). This increases the risk for sarcopenia and fracture, and such fractures are a leading driver of morbidity and mortality in older adults (Cava et al., 2017; Center et al., 1999). We recently published the primary outcomes of the Incorporating Nutrition, Vests, Education, and Strength Training (INVEST) in Bone Health randomized controlled trial (Beavers et al., 2025). INVEST in Bone Health aimed to test whether wearing a weighted vest during weight loss better preserved bone health compared to weight loss alone, and whether this was comparable to weight loss paired with structured resistance training—a health behavior often associated with improved bone health and quality of life (Daly et al., 2005; Villareal et al., 2017; Armamento-Villareal et al., 2020; Pi et al., 2018; Physical Activity Guidelines Advisory Committee, 2018)—in older adults who were overweight or living with obesity. All groups lost similar amounts of body weight (9%–11%), but neither those wearing the vest nor those engaging in structured resistance training retained greater bone density relative to those in the weight loss-only condition.

One possible reason for the absence of a group difference may be that the bone-sparing benefits of the weighted vest may be moderated by the amount of time one spends standing and therefore exposed to additional loading. Put differently, those who wore a weighted vest and spent more time in non-sedentary postures (e.g., standing, ambulating) would be exposed to a greater volume of loading and therefore may demonstrate greater effect of the vest than those who spent less time in these postures. Early data suggest that greater time spent standing is associated with better BMD in those with spinal injury (Alekna et al., 2008), and more time spent sedentary is associated with poorer bone health in women (Chastin et al., 2014). A strong body of evidence underscores that *unloading* (e.g., during bed rest or space flight) rapidly and negatively affects the skeleton (LeBlanc et al., 2007), particularly in weight-bearing regions (Kohrt et al., 2009). To date, however, no data exist on the relationship between upright time and change in BMD among older adults engaged in weight loss with or without a weighted vest or resistance training. Herein we examine whether objectively monitored time spent upright moderates the relationship between group assignment and change in bone mineral density within the INVEST in Bone Health trial. We hypothesized that those who received the weighted vest (WL+VEST) and engaged in more upright time would demonstrate better hip BMD. By contrast, given the absence of a consistent external loading stimulus across the day, we hypothesized that those in the weight loss-only or weight loss plus resistance training conditions would not demonstrate a significant relationship between upright time and BMD.

## 2 Methods

### 2.1 Study design

The protocol for the INVEST in Bone Health trial has been described elsewhere (Miller et al., 2021). In brief, INVEST in Bone Health was a 12-month randomized controlled trial conducted from September 2019 through April 2024. Participants were randomly assigned in a 1:1:1 fashion to engage in dietary weight loss alone (WL), weight loss plus resistance training (WL+RT), or weight loss plus weighted vest use (WL+VEST). All study procedures were approved by the Wake Forest University School of Medicine institutional review board (IRB# 00058279), and all participants provided written informed consent.

### 2.2 Participants

Participants were community-dwelling older adults (aged 60–85 years) who were weight stable (no WL >5% within the previous 6 months) and living with obesity [body mass index (BMI): 30–40 kg/m^2^)] or who were both overweight (BMI: 27 - <30 kg/m^2^) and had at least one obesity-related comorbidity and willing to engage in study procedures. Interested individuals were not eligible if they engaged in regular resistance and/or aerobic exercise for >60 min on> 5 days per week over the prior 6 months, had severe cardiometabolic disease, musculoskeletal impairments that precluded weighted vest use or exercise training, cognitive impairment, or used a prescription osteoporosis or WL medication within the previous year. In-depth characteristics of the sample are available in a prior publication (Beavers et al., 2025).

### 2.3 Interventions

Participants (N = 150 recruited between July 2020 and February 2023) were randomly assigned using random permuted blocks of size 3, 6, 9, and 12 and stratified by sex to one of three groups: WL, WL+VEST, or WL+RT. The WL intervention involved the provision of a nutritionally complete partial meal replacement program by Medifast, Inc. (the “4 & 2 & 1 Plan®”) with the aim of inducing a 10% loss of initial body mass within 12 months. Participants in all conditions engaged in group nutrition classes (weekly for the first 6 months, biweekly for the latter 6 months) that were designed to provide social support, education on nutrition concepts, and practice in dietary behavior self-regulation strategies. Those in the WL+VEST group were asked to wear a weighted vest for a target duration of 8 h per day during the most active part of the day. The vest was initially unloaded and then adjusted weekly based on the total amount of weight lost using 1/8th-pound blocks with a maximum load of 10% of initial body mass. On average, 78% ± 30% of the weight that was lost was replaced in the vest (Beavers et al., 2025). Participants were asked to keep a daily log of wear time, vest load, and any complications associated with wear. As reported previously, average self-reported daily wear time across the 12 months was 7.1 ± 1.5 h per day (Beavers et al., 2025). Of relevance to this analysis, participants received no specific instructions on modifying their daily sitting or standing behaviors. Finally, those in the WL+RT condition attended a supervised and center-based progressive RT program on three non-consecutive days each week. Participants aimed to complete three sets of 10–12 repetitions of eight upper- and lower-body exercises at 70%–75% of their one repetition max.

### 2.4 Measures

Participants self-reported key demographic characteristics, including age and sex, at baseline. BMD at the hip was a focal outcome of the INVEST in Bone Health trial. The primary outcome of the trial was 12-month change in quantitative computed tomography (CT)-acquired total hip trabecular volumetric bone mineral density (vBMD); accordingly, these measures were collected at baseline and months 6 and 12. Total hip integral (hereafter referred to simply as “total hip”) and cortical vBMD were also collected at each time point. In addition to CT, participants completed dual energy x-ray absorptiometry (DXA) assessments at baseline, 6 months, and 12 months. From this, a measure of total hip areal bone mineral density (aBMD) was extracted, as was aBMD of the femoral neck and lumbar spine. To compute BMI at each time point, height was measured without shoes to the nearest 0.1 cm via stadiometer, and body mass was recorded using a calibrated and certified balance beam scale to the nearest 0.1 kg. Finally, to measure posture, participants wore the ActivPAL 4 accelerometer (PAL Technologies, Glasgow, Scotland) on the midline of their non-dominant thigh for 7 days at each time point (baseline, 6 months, 12 months). The ActivPAL 4 provides an excellent classification of sedentary postures, standing, and stepping behaviors (O'Brien et al., 2022). In this study we summed daily time spent standing and stepping (i.e., time spent upright) to provide insight on total time spent experiencing the load from the vest. ActivPAL data were downloaded via PALBatch version 9 following a 24-h wear protocol and behaviors were classified via the CREA version 1.3 algorithm. Data were averaged across valid days (i.e., days with at least 20 h of wear time as is standard for the ActivPAL 24-h wear protocol) at a given time point, and at least 3 valid days were required for inclusion in analyses.

### 2.5 Analyses

Baseline descriptive statistics [i.e., mean (standard deviation) for continuous variables, n (%) for count variables] were computed for the sample overall and for each group. To investigate whether time spent upright moderated the impact of the intervention on BMD, we fit a series of linear mixed models, each including a participant-specific random intercept. For each model, change in BMD [computed as change in aBMD (mg/m^2^) or vBMD (mg/m^3^) from baseline to 6 or 12 months] was included as the dependent variable and predictors included the baseline BMD value, visit (6 or 12 months), group, and average daily upright duration. Additionally, models included the interaction between upright duration and group to give insight into whether the relationship between group assignment and change in BMD differed by the amount of time participants spent upright at a given time point. Additional covariates included BMI (to investigate the relationship between upright duration and BMD independent of body mass), age, and sex. Interactions were retained in the model at *p* < 0.10 as determined by marginal omnibus test. Significant interactions were examined via plots of fixed predicted values and *post hoc* tests contrasting estimated marginal trends between groups. Additionally, to aid in interpretation, coefficients involving upright time were multiplied by 30 to represent a 30-min difference in average daily upright duration. Statistical significance was established at *p* < 0.05 (note that as an exploratory analysis, no adjustment for multiple comparisons was employed). To investigate the influence of age and sex on each model, we conducted a final set of sensitivity analyses without these covariates. Their removal did not influence our results nor interpretation. All analyses were completed in R version 4.5.1 (R Core Team, 2021).

## 3 Results

### 3.1 Participant characteristics

Participant descriptive statistics are displayed in Table 1. A total of 150 participants completed the parent trial (see the primary outcomes manuscript (Beavers et al., 2025) for a description of the complete sample). Of these, 134 (WL n = 43, WL+RT n = 45, WL+VEST n = 46; 67 ± 4.8 years of age) had sufficient data for inclusion in DXA and/or CT models. Of these participants, 131 had sufficient DXA data for inclusion (WL n = 41; WL+RT n = 45; WL+VEST n = 45) and 132 participants had sufficient CT data for inclusion in analysis (WL n = 43, WL+RT n = 44, WL+VEST n = 45; see Supplementary Tables S1 and S2 for participant characteristics). Those without sufficient data for inclusion in DXA models (n = 3) were 70.66 ± 2.96 years of age; two were female and one was male; one identified as Black American, one as White, and one as more than one race; one had a college education and two achieved a post-graduate education; and had a BMI of 35.67 ± 2.15 kg/m (Villareal et al., 2005). None had sufficient baseline accelerometer data to derive baseline upright time (notably, all included participants had sufficient accelerometer data at later timepoints to be included in analyses). Those without sufficient data for inclusion in CT models (n = 2) were 68.74 ± 5.98 years of age; both were female and White; one had a college education and the other a post-graduate education; had a BMI of 33.05 ± 2.33 kg/m^2^ and spent 251.61 ± 15.68 min upright at baseline.

**TABLE 1:** Overall participant characteristics for those with sufficient data for inclusion in DXA and/or CT models.

Variable	WL	WL+RT	WL+VEST	Overall
	(N = 43)	(N = 45)	(N = 46)	(N = 134)
Age (years)	66.5 (4.4)	66.9 (5.3)	67.5 (4.6)	67.0 (4.8)
Sex				
Female	30 (69.8%)	34 (75.6%)	33 (71.7%)	97 (72.4%)
Race				
Black American	7 (16.3%)	14 (31.1%)	13 (28.3%)	34 (25.4%)
White	34 (79.1%)	31 (68.9%)	32 (69.6%)	97 (72.4%)
More than one	2 (4.7%)	0 (0%)	1 (2.2%)	3 (2.2%)
Education				
High school	8 (18.6%)	7 (15.6%)	13 (28.3%)	28 (20.9%)
College	25 (58.1%)	24 (53.3%)	20 (43.5%)	69 (51.5%)
Post-graduate	10 (23.3%)	14 (31.1%)	13 (28.3%)	37 (27.6%)
BMI (kg/m^2^)	33.3 (3.3)	33.7 (3.0)	33.8 (3.6)	33.6 (3.3)
Upright time (min)	314.1 (99.0)^b^	331.0 (100.6)	314.7 (100.2)^a^	320.1 (99.5)^c^
Steps	6499.1 (2500.0)^b^	6912.5 (2729.4)	6306.2 (2602.2)^a^	6574.9 (2608.2)^c^
Total hip aBMD (mg/cm^2^)	1012.3 (138.8)^b^	1033.1 (145.2)	1024.8 (127.2)^a^	1023.7 (136.5)^c^
Femoral neck aBMD (mg/cm^2^)	953.3 (138.8)^b^	965.4 (127.9)	942.4 (147.0)^a^	953.7 (137.4)^c^
Spine aBMD (mg/cm^2^)	1236.3 (188.8)^b^	1318.8 (272.8)	1280.2 (186.9)^a^	1279.7 (221.6)^c^
Total integral vBMD (mg/cm^3^)	288.7 (40.9)	298.5 (43.2)^a^	292.7 (40.5)^a^	293.3 (41.4)^b^
Trabecular vBMD (mg/cm^3^)	129.1 (19.4)	135.1 (21.5)^a^	131.2 (21.6)^a^	131.8 (20.9)^b^
Cortical vBMD (mg/cm^3^)	697.5 (30.2)	700.4 (28.7)^a^	704.7 (23.6)^a^	700.9 (27.5)^b^

WL, weight loss; WL+RT, weight loss plus resistance training; WL+VEST, weight loss plus weighted vest; kg, kilograms; m, meters; aBMD, areal bone mineral density; vBMD, volumetric bone mineral density; mg, milligrams; cm, centimeters; min, minutes. ^a^n = 1 missing; ^b^n = 2 missing, ^c^n = 3 missing.

### 3.2 Results for dual energy x-ray absorptiometry-assessed areal bone mineral density

**FIGURE 1:**
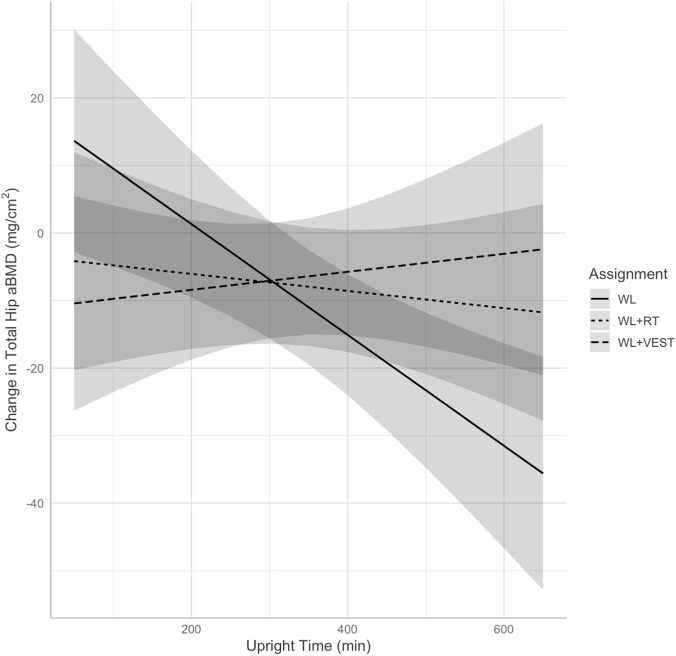
Predicted change in total hip aBMD (mg/cm^2^) at 6 and 12 months illustrating the interaction between group assignment and time spent upright as measured via accelerometer. WL, weight loss; WL+RT, weight loss plus resistance training; WL+VEST, weight loss plus weighted vest; aBMD, areal bone mineral density.

Fixed effects from mixed models for DXA variables can be found in Table 2. The model for total hip aBMD revealed a significant interaction between group and time spent upright (*p* = 0.023), and *post hoc* contrasts revealed this was driven by a significant difference in the relationship between average daily upright time and change in total hip aBMD between WL and WL+VEST (*p* = 0.009) and between WL and WL+RT (*p* = 0.043) but not between WL+RT and WL+VEST (*p* = 0.450). Predicted associations between upright time and change in total hip aBMD are displayed in Figure 1. Investigation of estimated marginal trends revealed a significant negative association between average daily upright time and aBMD within WL (B = −0.08, *p* = 0.001) suggesting that a 30-min higher average duration of upright time was associated with a 2.4 mg/cm^2^ decrease in total hip aBMD. By contrast, there was no significant association between upright time and hip aBMD in the WL+VEST group (B = 0.013, *p* = 0.603) or WL+RT (B = −0.013, *p* = 0.584) condition. Put differently, a 30-min higher average duration of upright time was associated with a 0.39 mg/cm^2^ increase in total hip aBMD for WL+VEST, and a 0.39 mg/cm^2^ reduction in total hip aBMD for WL+RT.

**TABLE 2:** Fixed effects from mixed effects models.

Variable	DXA (aBMD, mg/cm^2^)								
	Total hip			Femoral neck			Spine		
	B	95% CI	*p*	B	95% CI	*p*	B	95% CI	*p*
Intercept	−22.96	(−88.94, 43.03)	0.492	−4.36	(−98.95, 90.24)	0.928	−110.75	(−241.96, 20.47)	0.097
Baseline value	0.01	(−0.02, 0.03)	0.741	−0.03	(−0.07, 0.01)	0.144	0.01	(−0.03, 0.04)	0.746
BMI	0.99	(−0.07, 2.04)	0.067	0.83	(−0.74, 2.41)	0.295	2.79	(0.53, 5.05)	**0.016**
Visit (ref: month 6)	−7.80	(−10.82, −4.77)	**<0.001**	−5.52	(−10.39, −0.65)	**0.027**	−4.73	(−11.13, 1.68)	0.146
Age	0.09	(−0.64, 0.83)	0.803	0.39	(−0.69, 1.47)	0.476	0.48	(−1.12, 2.08)	0.553
Sex (ref: Male)	0.10	(−8.35, 8.55)	0.982	−9.78	(−21.95, 2.38)	0.114	−10.63	(−28.79, 7.53)	0.249
Group*	--	--	0.055	--	--	0.586	--	--	0.305
Upright time	−0.08	(−0.13, −0.03)	**0.001**	−0.04	(−0.08, 0.00)	0.065	0.01	(−0.05, 0.07)	0.737
Group x upright time*	--	--	**0.023**	--	--	--	--	--	--

WL, weight loss; WL+RT, weight loss plus resistance training; WL+VEST, weight loss plus weighted vest; aBMD, areal bone mineral density; mg, milligrams; cm, centimeter; BMI, body mass index. *Significance value obtained from marginal omnibus test. Bolded values are statistically significant.

Models did not reveal significant interactions between group and upright time for aBMD at the femoral neck or spine (*p* = 0.158 and *p* = 0.109 respectively). For femoral neck aBMD, there was a significant main effect for visit [B = −5.52; *p* = 0.027; 95% CI (−10.39, −0.65)], such that aBMD decreased by 5.52 mg/cm^2^ between months 6 and 12 after adjusting for baseline. The main effect for upright time approached statistical significance [B = −0.04; *p* = 0.065; 95% CI (−0.08, 0.00)] such that a 30-min higher average duration of upright time was associated with a 1.2 mg/cm^2^ reduction in femoral neck aBMD.

### 3.3 Results for quantitative computed tomography-measured volumetric bone mineral density

**FIGURE 2:**
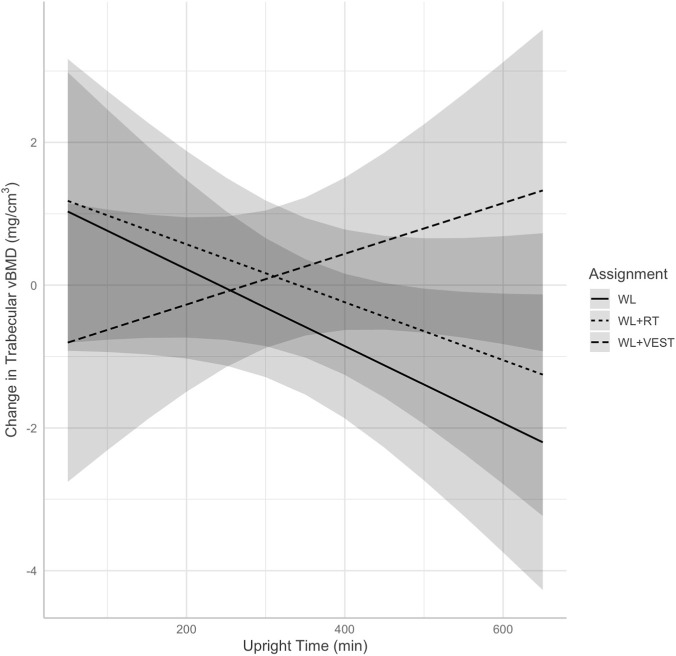
Predicted change in total hip trabecular vBMD (mg/cm^3^) at 6 and 12 months illustrating the interaction between group assignment and time spent upright as measured via accelerometer. WL, weight loss; WL+RT, weight loss plus resistance training; WL+VEST, weight loss plus weighted vest; vBMD, volumetric bone mineral density.

The fixed effects from mixed models for CT variables can be found in Table 3. The model for integral vBMD did not reveal a significant group by upright time interaction (*p* = 0.601), and therefore the interaction was not retained in the final model. There was a significant main effect for time [B = −2.88, *p* < 0.001, 95% CI (−3.80, −1.97)] such that integral vBMD decreased by 2.88 mg/cm^3^ between months 6 and 12 after controlling for baseline. Regarding trabecular vBMD, the mixed model revealed an interaction between group assignment and time spent upright was retained in the final model (*p* = 0.094). Exploratory *post hoc* contrasts indicated this was driven by significantly different relationships between upright time and change in vBMD between the WL vs. WL+VEST conditions (*p* = 0.043); no other contrasts were significant (all *p* ≥ 0.080). This interaction is illustrated in Figure 2. Investigation of estimated marginal trends revealed a non-significant negative relationship between trabecular vBMD and average daily upright time among those in the WL condition (B = −0.005, *p* = 0.077), such that a 30-min higher duration of upright time was associated with a 0.15 mg/cm^3^ decrease in vBMD. Among those in the WL+VEST condition, a 30-min higher duration of upright time was associated with a non-significant 0.12 mg/cm^3^ increase in vBMD (B = 0.004, *p* = 0.267). There was also a significant main effect for visit [B = −1.47; *p* < 0.001; 95% CI (−1.87, −1.07)] such that trabecular vBMD decreased by 1.47 mg/cm^3^ between months 6 and 12 after controlling for baseline. Note that fitting the model without the interaction did not meaningfully change this interpretation (i.e., the main effect for visit was retained, no other relationships were significant). Finally, there was no group by upright time interaction for cortical vBMD of the hip (*p* = 0.480), nor were there any significant main effects of interest in the model without the interaction.

**TABLE 3:** Fixed effects from mixed effects models.

Variable	CT hip (vBMD, mg/cm^3^)								
	Total			Trabecular			Cortical		
	B	95% CI	*p*	B	95% CI	*p*	*B*	95% CI	*p*
Intercept	18.36	(−3.01, 39.73)	0.092	−0.59	(−7.96, 6.79)	0.875	144.45	(83.06, 205.85)	**<0.001**
Baseline value	0.01	(−0.02, 0.04)	0.428	−0.01	(−0.03, 0.01)	0.390	−0.15	(−0.22, −0.07)	**<0.001**
BMI	0.00	(−0.35, 0.35)	0.983	0.02	(−0.1, 0.15)	0.715	−0.45	(−1.03, 0.13)	0.128
Visit (ref: month 6)	−2.88	(−3.8, −1.97)	**<0.001**	−1.47	(−1.87, −1.07)	**<0.001**	−0.01	(−1.42, 1.4)	0.991
Age	−0.28	(−0.53, −0.02)	**0.033**	0.03	(−0.05, 0.12)	0.423	−0.29	(−0.72, 0.14)	0.180
Sex (ref: Male)	−1.64	(−4.34, 1.06)	0.231	−0.75	(−1.66, 0.17)	0.108	−3.22	(−7.8, 1.36)	0.166
Group*	--	--	0.402	--	--	0.208	--	--	0.827
Upright time	0.00	(−0.01, 0.01)	0.753	−0.01	(−0.01, 0.00)	0.077	0.00	(−0.01, 0.02)	0.861
Group x upright time*	--	--	--	--	--	0.094	--	--	--

WL, weight loss; WL+RT, weight loss plus resistance training; WL+VEST, weight loss plus weighted vest; vBMD, volumetric bone mineral density; mg, milligrams; cm, centimeter; BMI, body mass index. *Significance value obtained from marginal omnibus test. Bolded values are statistically significant.

## 4 Discussion

The main findings of the INVEST in Bone Health trial revealed that, on average, those who engaged in either a weighted vest program or resistance training program paired with dietary weight loss did not preserve bone mass to a greater extent than did those who engaged in dietary weight loss alone (Beavers et al., 2025). Herein we sought to explore whether time spent upright (i.e., standing, stepping) moderated the effect of the intervention on BMD as measured by both DXA and CT. We hypothesized that those who spent more time upright, and therefore spent more time exposed to the stimulus provided by the weighted vest, would demonstrate better preservation of BMD—especially in the hip—during a dietary weight loss program than those spending more time sedentary. We did not hypothesize a significant relationship between upright time and change in BMD among those in the WL+RT or WL conditions. Results partially supported our hypothesis, as there was a significant interaction between intervention assignment—specifically WL vs. either WL+VEST or WL+RT—and time spent upright on baseline-adjusted hip aBMD. Somewhat contrary to our hypothesis, this was driven by a negative relationship between average daily upright duration and total hip aBMD in the WL condition. For those in the WL condition, a 30-min higher average duration of upright time was associated with a statistically significant 2.4 mg/cm^2^ decrease in total hip aBMD. By contrast, for those in the WL+VEST or WL+RT conditions, a 30-min higher average duration of upright time was associated with a non-significant 0.39 mg/cm^2^ increase and a non-significant 0.39 mg/cm^2^ decrease in total hip aBMD, respectively. A similar relationship was observed for CT-derived trabecular vBMD, though this interaction did not achieve statistical significance.

The finding that greater time spent upright was associated with greater losses in BMD within the WL condition is intriguing, suggesting greater exposure to reduced body weight may signal skeletal adaptation as posited by the mechanostat hypothesis (Iwaniec and Turner, 2016). This hypothesis was originally coined by Frost in 1987 (Frost, 1987), proposing that mechanical loading is a key driver of bone biology. Specifically, cells within the bone detect external loading—which may be increased or sustained via external load or decreased in response to weight loss—and in turn regulate modeling and remodeling responses. It is also notable that those randomized to receive RT demonstrated a neutral relationship between upright time and change in DXA-derived hip aBMD, though this relationship was not present for CT-derived hip vBMD. There is some evidence that exposure to greater loading due to resistance training can help to mitigate BMD loss during weight loss, though meta-analyses report that these effects are somewhat inconsistent (Mesinovic et al., 2021). Still, the observation that RT appeared to neutralize any relationship between upright time and change in aBMD is worth further investigation. Finally, we did not observe any such interaction for spine aBMD; an unsurprising finding as the hip tends to be more responsive than other sites to changes in body mass (Soltani et al., 2016).

These exploratory findings are relevant to those interested in the effect of weighted vest use on bone health in older adults undergoing weight loss. They suggest that, in addition to behavioral programming targeting daily vest use, interventions should likewise aim to increase the time individuals spend standing or stepping. Fortunately, the last decade has seen a rapid growth in interventions targeting this precise behavioral outcome out of an interest in promoting either the reduction of sitting time or the accumulation of physical activity. Indeed, our research group has developed one such intervention that focuses on combining dietary weight loss with *daylong* movement, using a combination of remotely delivered group behavioral intervention strategies and digital health tools to promote frequent participation in bodily movement (Fanning et al., 2018a; Fanning et al., 2021; Fanning et al., 2020; Fanning et al., 2022a; Fanning et al., 2025; Fanning et al., 2022b; Fanning et al., 2018b). There are several benefits to such an approach from a weight loss perspective. Relative to traditional modes of exercise, participants are encouraged to identify an array of enjoyable modes of activity, often comprising a combination of domains of activity (e.g., leisure time exercise, household activity, transport activity) to account for changing daily time-use demands, seasonality, and personal preferences. Moving often helps to reduce hedonic eating, and individuals learn to conceptualize movement as a behavior that occurs across the day rather than in a single bout, therefore reducing compensatory reductions in non-exercise physical activity and increases in sedentary behavior that often accompany initiation of structured exercise (Melanson, 2017). We have demonstrated that older adults engaging in a daylong movement and weight loss program lose similar weight in the short term but maintain weight loss better in the long term relative to those who engage in weight loss and structured treadmill exercise (Fanning et al., 2022b). Notably, the activities participants engage in tend to be aerobic and ambulatory in nature. Incorporating weighted vests aligns well with the spirit of a daylong movement intervention in that it integrates resistance training into daily life. The results presented herein also suggest that use of a weighted vest may help to preserve bone mass during weight loss. Future work is warranted examining the acceptability and efficacy of a combination of weighted vest, weight loss, and daylong movement for reducing body mass, sustaining bone health, and enhancing physical activity volume.

### 4.1 Strengths and limitations

There are several notable strengths to this secondary analysis. For instance, INVEST in Bone Health was a novel randomized controlled trial that allowed for comparisons of dietary weight loss alone or paired with either weighted vest user or resistance training. Additionally, upright time was quantified objectively using the ActivPAL accelerometer, which provides high-quality classification of posture and avoids bias associated with self-report (Prince et al., 2020; Schaller et al., 2016). There are also several notable limitations. As a secondary analysis, we did not conduct an *a priori* power analysis and therefore it may be that there was insufficient power to detect significant interactions between time spent upright and group assignment on CT-derived measures of BMD. Additionally, the sample was comprised of primarily female, White, and college-educated participants. As these factors are associated with different daily time-use patterns (e.g., males, individuals who do not identify as White, and those with lower levels of education engage in higher levels of occupational physical activity) (Gudnadottir et al., 2019), it would be beneficial to investigate the impact of weighted vest use on individuals belonging to more varied sociodemographic groups and living in more diverse environments. Finally, future work would benefit from the development of objective markers of vest use (e.g., sensor-derived vest wear) to facilitate examination of the amount and duration of daily vest use while in an upright posture required to sustain BMD during weight loss.

## 5 Conclusion

Managing the impact of obesity on health and quality of life during aging is a persistent public health challenge. Reducing body mass is associated with a host of health benefits but also brings about losses in fat-free mass that may predispose an individual to sarcopenia and increased fracture risk through loss of lean mass and bone mass. Exploratory findings suggest older adults who engaged in dietary weight loss while wearing a weighted vest sustained better bone mineral density if they spent more of their day standing or stepping. These results point to the need for additional research investigating the efficacy of a weighted vest intervention paired with daylong movement for sustaining bone health among older adults as they lose weight.

## Data Availability

The raw data supporting the conclusions of this article will be made available by the authors, without undue reservation.
