# Supplementary material for: Does time spent upright moderate the influence of a weighted vest on change in bone mineral density during weight loss among older adults? A secondary analysis of the INVEST in bone health randomized controlled trial
Source: Front Aging. 2026 Feb 11;7:1729001. doi: 10.3389/fragi.2026.1729001 (PMC12932580; doi:10.3389/fragi.2026.1729001)
Supplement: Supplementary file 1 [file Table1.docx]

Supplementary Material

# Supplementary Tables

|  | **DXA** | | | |  |
| --- | --- | --- | --- | --- | --- |
|  | **WL** | **WL+RT** | **WL+VEST** | **Overall** | |
|  | **(N=41)** | **(N=45)** | **(N=45)** | **(N=131)** | |
| **Age (years)** | 66.3 (4.4) | 66.9 (5.3) | 67.3 (4.6) | 66.9 (4.8) | |
| **Sex** |  |  |  |  | |
| Female | 29 (70.7%) | 34 (75.6%) | 32 (71.1%) | 95 (72.5%) | |
| **Race** |  |  |  |  | |
| Black American | 6 (14.6%) | 14 (31.1%) | 13 (28.9%) | 33 (25.2%) | |
| White | 34 (82.9%) | 31 (68.9%) | 31 (68.9%) | 96 (73.3%) | |
| More than One | 1 (2.4%) | 0 (0%) | 1 (2.2%) | 2 (1.5%) | |
| **Education** |  |  |  |  | |
| High School | 8 (19.5%) | 7 (15.6%) | 13 (28.9%) | 28 (21.4%) | |
| College | 24 (58.5%) | 24 (53.3%) | 20 (44.4%) | 68 (51.9%) | |
| Post-Graduate | 9 (22.0%) | 14 (31.1%) | 12 (26.7%) | 35 (26.7%) | |
| **BMI (kg/m^2^)** | 33.1 (3.3) | 33.7 (3.0) | 33.8 (3.7) | 33.6 (3.3) | |
| **Upright Time (min)** | 314.1 (99.0) | 331.0 (100.6) | 314.7 (100.2) | 320.1 (99.5) | |

**Supplemental Table 1.** Participant characteristics for those with sufficient data for inclusion in DXA models. WL = weight loss, WL+RT = weight loss plus resistance training, WL+VEST = weight loss plus weighted vest, kg = kilograms, m = meters, min = minutes

|  | **CT** | | | | |
| --- | --- | --- | --- | --- | --- |
|  | **WL** | **WL+RT** | **WL+VEST** | **Overall** |  |
|  | **(N=43)** | **(N=44)** | **(N=45)** | **(N=132)** |  |
| **Age (years)** | 66.5 (4.4) | 67.0 (5.4) | 67.3 (4.6) | 66.9 (4.8) |  |
| **Sex** |  |  |  |  |  |
| Female | 30 (69.8%) | 33 (75.0%) | 32 (71.1%) | 95 (72.0%) |  |
| **Race** |  |  |  |  |  |
| Black American | 7 (16.3%) | 14 (31.8%) | 13 (28.9%) | 34 (25.8%) |  |
| White | 34 (79.1%) | 30 (68.2%) | 31 (68.9%) | 95 (72.0%) |  |
| More than One | 2 (4.7%) | 0 (0%) | 1 (2.2%) | 3 (2.3%) |  |
| **Education** |  |  |  |  |  |
| High School | 8 (18.6%) | 7 (15.9%) | 13 (28.9%) | 28 (21.2%) |  |
| College | 25 (58.1%) | 24 (54.5%) | 19 (42.2%) | 68 (51.5%) |  |
| Post-Graduate | 10 (23.3%) | 13 (29.5%) | 13 (28.9%) | 36 (27.3%) |  |
| **BMI (kg/m^2^)** | 33.3 (3.3) | 33.8 (3.0) | 33.8 (3.7) | 33.6 (3.3) |  |
| **Upright Time (min)** | 314.1 (99.0)^b^ | 332.5 (101.3) | 316.4 (100.7)^a^ | 321.1 (99.9)^c^ |  |

**Supplemental Table 2.** Participant characteristics for those with sufficient data for inclusion in CT models. WL = weight loss, WL+RT = weight loss plus resistance training, WL+VEST = weight loss plus weighted vest, kg = kilograms, m = meters, min= minutes. ^a^n=1 missing; ^b^n=2 missing, ^c^n=3 missing
